# Efficacy, safety and drug survival of thioguanine as maintenance treatment for inflammatory bowel disease: a retrospective multi-centre study in the United Kingdom

**DOI:** 10.1186/s12876-020-01441-6

**Published:** 2020-09-11

**Authors:** Ahmed B. Bayoumy, Elsa L. S. A. van Liere, Melek Simsek, Ben Warner, Aathavan Loganayagam, Jeremy D. Sanderson, Simon Anderson, Jonathan Nolan, Nanne K. de Boer, Chris J. J. Mulder, Azhar Ansari

**Affiliations:** 1 Department of Gastroenterology and Hepatology, Amsterdam UMC, VU University Medical Centre, Amsterdam, The Netherlands; 2 Department of Gastroenterology, Guy’s and St Thomas’ NHS Foundation Trust, London, UK; 3 Department of Gastroenterology, Queen Elizabeth Hospital, Woolwich, UK; 4 Department of Gastroenterology, Surrey and Sussex NHS, Easy Surrey Hospital, Surrey, UK; 5 Department of Gastroenterology and Hepatology, Amsterdam UMC, VU University Medical Centre, AG&M Research Institute, Amsterdam, The Netherlands

**Keywords:** Thioguanine, Thiopurines, IBD, Crohn’s disease, Ulcerative colitis, Drug repositioning, Drug survival

## Abstract

**Background:**

Thioguanine (TG) is a thiopurine which has been used for patients with inflammatory bowel disease (IBD), who have failed azathioprine (AZA) or mercaptopurine (MP) due to adverse events or suboptimal response. Its widespread use has been hampered due to concerns about nodular regenerative hyperplasia (NRH) of the liver. The aim of this study was to investigate the long-term efficacy and safety of low-dose TG therapy in IBD patients failing AZA and MP.

**Methods:**

A retrospective multicentre study was performed in IBD patients who failed prior treatment with conventional thiopurines with or without following immunomodulation (thiopurine-allopurinol, biologicals, methotrexate, tacrolimus) and were subsequently treated with TG as rescue monotherapy between 2003 and 2019 at three hospitals in the United Kingdom. Clinical response, adverse events, laboratory results, imaging and liver biopsies were retrospectively collected.

**Results:**

A total of 193 patients (57% female and 64% Crohn’s disease) were included, with a median daily TG dose of 20 mg (range: 20–40 mg), a median treatment duration of 23 months (IQR 10–47) and a median follow-up of 36 months (IQR 22–53). The clinical response rate at 12 months was 65 and 54% remained on TG until the end of follow-up. Adverse events consisted primarily of elevated liver tests (6%), myelotoxicity (7%) and rash (5%). NRH was histologically diagnosed in two patients and two other patients (1%) developed non-cirrhotic portal hypertension. The median 6-TGN and TPMT levels were 953 pmol/8 × 10^5^ RBC (IQR 145–1761) and 47 mu/L (IQR 34.5–96).

**Conclusions:**

Long-term follow-up suggests that TG can be an effective and well-tolerated therapy in more than half of difficult-to-treat and multi-therapy failing IBD patients. Findings of this study indicate that TG can be used safely and the occurrence of hepatotoxicity was low. The incidence rate of NRH was within the background incidence.

## Background

The classic thiopurines mercaptopurine (MP), and its licenced and more widely used prodrug azathioprine (AZA), are still first line immunosuppressives for treating inflammatory bowel diseases (IBD). In recent times, their role is increasingly being questioned because of their relative poor performance, mainly due to treatment and dosage limiting adverse events, when compared to biologics [1–3]. Nonetheless, a sizable subgroup of individuals experience a positive response (30–50%) with meaningful clinical outcomes such as sustained steroid sparing/ free remission combined with a reduced need for hospitalisation, surgery and biologic agents [4–10]. There is an emerging interest in small molecules, as they have advantages over biologicals (e.g. oral administration, lower costs, no antibody formation and therefore sustained efficacy) [11]. The thiopurines are well-established classical small molecules and hence share these benefits. However, the conventional thiopurines (AZA/MP) have been well described and accepted shortcomings include slow onset of action, relatively high rate of adverse events and subsequent frequent need for monitoring and/or dose optimisation. These time-consuming and often distressing problems have resulted in an increase of early use of biological therapy [12, 13].

Thioguanine (TG) was introduced in 1950 as treatment for leukaemia and about five decades later, clinicians started using it for treating IBD. Modern cases series suggest that TG offers better outcomes in terms of efficacy (51–60%) as rescue therapy, low incidence of adverse events and possibly a more rapid onset of action compared to conventional thiopurines [14–16]. However, its widespread use has been hampered due to concerns about hepatotoxicity (nodular regenerative hyperplasia [NRH]) but this is thought to be dose-dependent [17–19]. Other long-term risks of TG might be the development of lymphoma and skin malignancy, as has been shown with AZA/MP and anti-TNF therapies [20, 21].

Having considered these potential risks, the benefits of TG remain important in the treatment of patients with IBD [22]. For this reason, a few centres continue to use TG but only at a lowered dose (10–40 mg/day). Reports of outcomes from these centres in the United Kingdom (UK), The Netherlands and Australia confirm a good efficacy and lack of hepatotoxicity [14, 15, 23–25]. Moreover, in the Netherlands, TG has been provisional licenced for IBD since a few years [26, 27]. At the moment an estimated 6000 Dutch IBD use TG as rescue treatment for IBD [28].

For the reasons outlined, it is important to evaluate the long-term outcomes of a low dose TG approach. The aim of this study was to investigate the long-term efficacy and safety of low dose TG in a large IBD cohort failing conventional thiopurines, derived from three UK centres.

## Methods

### Study design and patient population

A retrospective, multicentre cohort study was performed in three centres in the UK (St. Thomas’ Hospital, Queen Elizabeth Hospital and East Surrey Hospital). Patients were identified by using local hospital pharmacy dispensing records dating from 2003 to 2019, as TG is only dispensed through the hospital pharmacy. Patients were included if they were diagnosed with Crohn’s Disease (CD), ulcerative colitis (UC) or IBD-unclassified (IBD-U) according to clinical, endoscopic and/or histological criteria and if they were treated with TG, either as monotherapy or concomitant therapy. Montreal classification were used to classify the IBD patients [29]. Patients without complete documented follow-up were excluded.

### Data collection

Patient and disease characteristics, drug history and clinical, biochemical, radiological and histopathological data were reviewed using the patients’ medical records. Laboratory data were taken at 3 months prior to TG initiation, at 6 months after initiation and at final follow-up. Laboratory parameters included haemoglobin, white blood cell count (WBC), platelet count, aspartate aminotransferase (AST), alanine aminotransferase (ALT), alkaline phosphatase (ALP), gamma glutamyl-transferase (GGT), bilirubin, albumin and 6-thioguanine nucleotides (6-TGNs). Most recent abdominal ultrasonography, magnetic resonance imaging (MRI) and liver biopsies were assessed.

### Safety assessment

Safety of TG therapy was assessed by evaluating 1) haematological parameters for myelotoxicity and NRH (through alkaline phosphatase) and 2) radiological, histopathological and biochemical parameters for liver toxicity. Histopathological liver biopsy reports were assessed and the pathologists’ findings and conclusions were used to assess the occurrence of NRH. Patients were categorized into ‘signs of NRH’ when minimal focal hyperplasia was present. Emphasis has been put on signs or findings of non-cirrhotic portal hypertension (NCPHT) (e.g. hepato- or splenomegaly, nodular abnormalities, ascites, varices). The occurrence of adverse events and reasons for withdrawal during the entire follow-up were recorded. Adverse events were defined as laboratory abnormalities and signs or symptoms that occurred after initiation of TG, and were listed according to the common terminology criteria for adverse events (CTCAE) (Version 5.0, released November 27, 2017) [30].

### Efficacy assessment

A positive clinical response was defined as: no (re)initiation of corticosteroids, (re)initiation of biological therapy or IBD-related surgery. CRP and ESR levels were collected to determine biochemical response. TG use at final follow-up was evaluated to assess long-term beneficial effect. Primary resistance was assigned when criteria for clinical effectiveness were not met within the first 6 months of therapy. Lastly, 6-TGN levels were collected to evaluate a possible correlation with clinical response.

### Statistical analysis

Data was presented as numbers with percentages, medians with interquartile range (IQR) or means with standard deviations. Depending on the kind of parameter, distribution, parametric or nonparametric tests including the Mann-Whitney U test, Wilcoxon signed-rank test, Kruskal Wallis, and the student t-test or chi-square test were used to test for differences within and between groups. A Kaplan-Meier plot was performed to assess the TG attrition rate. The survival curves were compared using the Mantel-Cox test. This study was reported according to the Strengthening the Reporting of Observational Studies in Epidemiology statement [31]. IBM SPSS Statistics V.25 was used for the statistical analysis. A *p*-value less than 0.05 was accepted as statistically significant.

### Ethical considerations

According to the guidelines of the UK Health Research Authority, as the data were collected as part of routine clinical care and were evaluated retrospectively, the study was considered a review of clinical practice and ethical approval was not required [32]. This study was conducted in accordance with the Declaration of Helsinki [33]. All data in this study was anonymised.

## Results

### Patient characteristics

In total, 209 IBD patients treated with TG were identified. Sixteen patients were excluded due to incomplete follow-up data, leading to a total number of 193 patients whom were included in the study. Of these, 110 patients were female (57%), 123 had CD (64%), 64 UC (33%) and 6 IBD-U (3%). Perianal disease was diagnosed in 23 patients (12%). Median age at IBD diagnosis and initiation of TG was 32 (IQR 21–44) and 43 years (IQR 33–56), respectively. Sixty-three patients (33%) had a history of bowel resection prior to TG therapy. Patient and disease characteristics are shown in Table 1.

**Table 1:** Patient and disease characteristics of the entire cohort (*n* = 193)

Variable	Results
**Male/female**	83/110
**Age at diagnosis, median (IQR)**	32 (21–44)
**Age at initiation TG, median (IQR)**	43 (33–56)
**Hospital**	
▪ **St. Thomas’ Hospital**	87 (45%)
▪ **East Surrey Hospital**	67 (35%)
▪ **Queen Elizabeth Hospital**	39 (20%)
**Crohn’s disease (Montreal classification)**	123 (64%)
**Age at diagnosis**	
▪ **< 17 (A1)**	16 (19%)
▪ **17–40 (A2)**	71 (58%)
▪ **> 40 (A3)**	29 (24%)
**Location**	
▪ **Ileal (L1)**	38 (31%)
▪ **Colonic (L2)**	15 (15%)
▪ **Ileocolonic (L3)**	62 (50%)
▪ **Upper gastrointestinal disease (L4)**	4 (3%)
**Behaviour**	
▪ **Nonstricturing, nonpenetrating (B1)**	45 (40%)
▪ **Stricturing (B2)**	36 (33%)
▪ **Penetrating (B3)**	26 (27%)
▪ **Perianal disease (p)**	23 (12%)
**Ulcerative colitis (Montreal classification)**	64 (33%)
**Extent**	
▪ **Proctitis (E1)**	18 (30%)
▪ **Left-sided (E2)**	24 (37%)
▪ **Pancolitis (E3)**	21 (33%)
**Severity**	
▪ **Asymptomatic (S0)**	9 (14%)
▪ **Mild (S1)**	15 (24%)
▪ **Moderate (S2)**	18 (28%)
▪ **Severe (S3)**	22 (34%)
**IBD unclassified**	6 (3%)
**Drug failure prior to thioguanine initiation**	193 (100%)
▪ **Azathioprine**	123 (58%)
▪ **Mercaptopurine**	11 (5%)
▪ **Azathioprine and mercaptopurine**	59 (27%)
▪ **Azathiopurine and allopurinol**	76 (39%)
▪ **Anti-tumour necrosis factor**	77 (40%)
▪ **Methotrexate**	36 (19%)
▪ **Tacrolimus**	8 (4%)
**Gastrointestinal surgery prior to thioguanine initiation**	63 (33%)
**Follow-up in months, median (IQR)**	36 (22–53)
**Treatment duration of thioguanine (months, IQR)**	23 (10–47)

Prior to TG initiation, AZA was used in 123 patients (58%), MP in 11 patients (5%) and both AZA and MP in 59 patients (27%). Seventy-six of 193 patients (39%) had used co-therapy with allopurinol to optimise AZA (AZA-Allo). The median daily dosages of AZA and MP were 100 mg (range: 75–200 mg) and 100 mg (range: 50–150). The dosage used for AZA-Allo was 50 mg for AZA and 100 mg for allopurinol. The most commonly reported reason for prior thiopurine failure was intolerance to thiopurines, which occurred in 150 patients (78%). Intolerances to thiopurines mostly recorded were nausea (22%), pancreatitis (21%) and arthralgia/myalgia (6%). Resistance to thiopurines occurred in 18 patients (9%). Twelve of those 18 resistant patients (67%) were primary non-responders. The rest of the patients were switched to TG for reasons such as patients’ preference, suboptimal 6-TGN levels and non-compliance.

Furthermore, 77 patients (40%) had a history of biological therapy (see Table 1). Sixty-one patients (79%) had loss of response on biologicals, six patients (8%) suffered from rash, five (6%) had infusion site reactions, two (3%) suffered from tonsillar abscess and three patients (4%) had psoriatic skin abnormalities.

The median daily dose for TG was 20 mg; 107 patients (55%) were treated with 20 mg once daily and 86 patients (45%) with 20 mg twice daily. Patients of East Surrey Hospital were commenced on 20 mg twice daily and after 6 months the lowest effective dose was achieved by reducing to 20 mg/day. At St. Thomas’ Hospital most patients were started on 20 mg once daily although some received 40 mg/day (20 mg twice daily) with the desire to reduce to 20 mg once daily if response allowed. At Queen Elizabeth Hospital, all patients received 20 mg once daily. The median treatment duration of TG was 23 months (IQR 10–47) and the median follow-up time of 36 months (IQR 22–53). Thirty-three patients (18%) started TG along with biological therapy; adalimumab (40 mg, two weekly) was started in 18 patients, infliximab (5 mg/kg, eight weekly) in 13 patients, ustekinumab (90 mg, eight weekly) in one patient and vedolizumab in one patient (300 mg, eight weekly). The median duration of follow-up was 12 months (IQR 7–15), in which 22 out of 33 patients (67%) were still using biologicals along with TG. The reasons to stop biologicals were due to loss of response and side-effects (i.e. infusion reactions).

### Efficacy

**Fig. 1:**
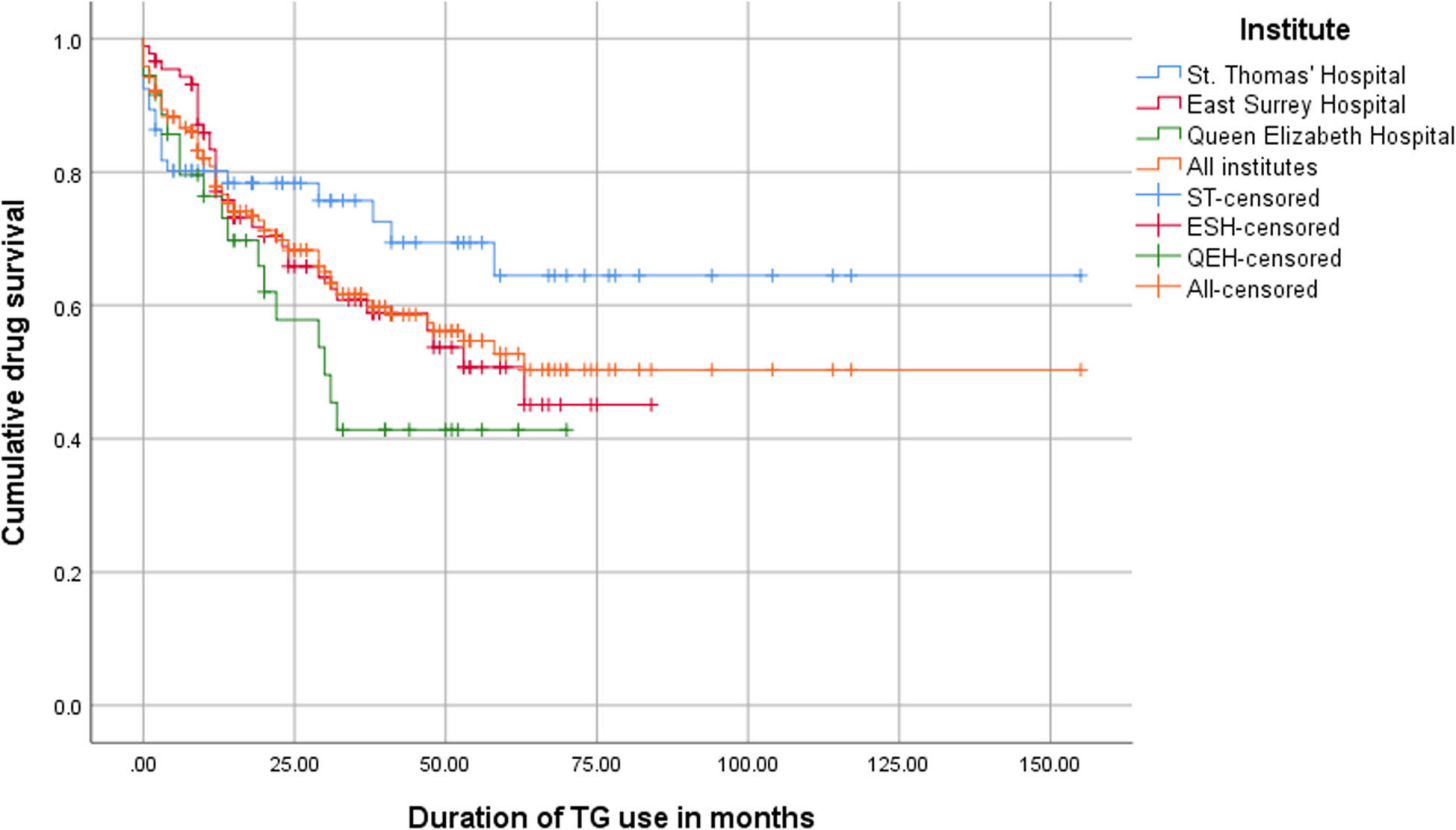
Drug survival of TG in 193 patients, showing the time to TG withdrawal/censoring in months per hospital. Median treatment duration of TG was 23 months. The differences between the curves were not significantly different (*p* = 0.12, Mantel-Cox test)

**Fig. 2:**
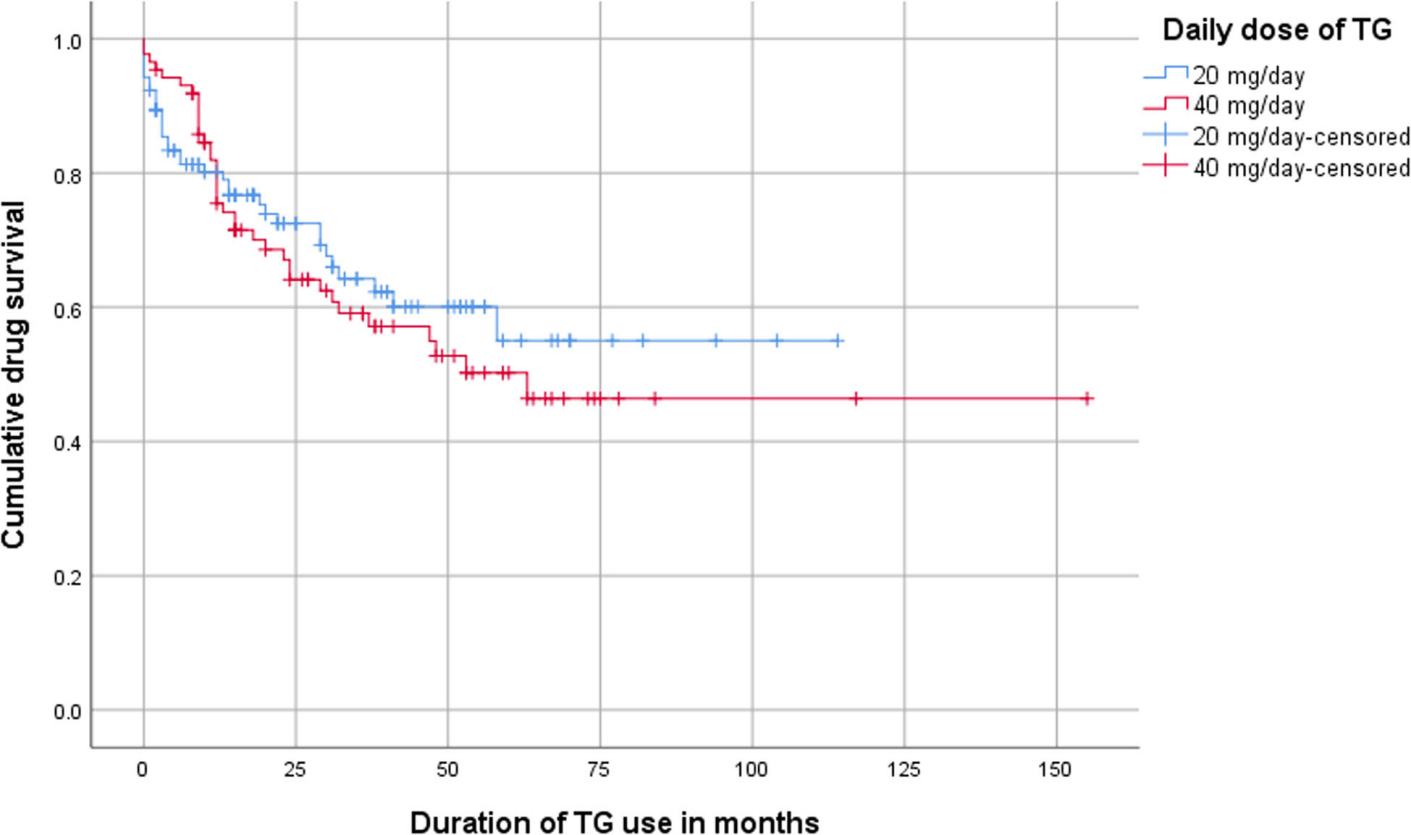
Drug survival curve of TG of patients receiving 20 mg TG per day compared to 40 mg/day. No significant differences were found between both curves (*p* = 0.57, Mantel-Cox test)

Clinical response at 6 and 12 months of TG therapy was seen in 71% (*n* = 137) and 65% (*n* = 125) of patients, respectively. The proportion of patients who continued TG therapy is depicted in a Kaplan-Meier survival curve (Fig. 1). Of the entire cohort, 153 (79%), 137 (71%) and 104 (54%) were still using TG at respectively 12 and 24 months after initiation, and at final follow-up. Drug survival did not depend on age, gender, type of IBD, bowel resection history, short bowel syndrome or IBD behavior. No statistical significant difference was observed between using a dosage of 20 or 40 mg per day (Fig. 2).

Thioguanine was discontinued in 89 patients (46%) during follow-up. Forty-three patients (22%) stopped TG due to intolerance, 15 (8%) due to resistance, 3 (2%) due to malignancy (melanoma, diffuse large B-cell lymphoma, recurrent breast cancer), 7 (4%) due to pregnancy, 4 (2%) due to non-compliance, 6 (3%) due to complete remission and 11 (5%) due to miscellaneous reasons. The median time till cessation of therapy due to intolerance or resistance was 9 (IQR 2–20) and 12 months (IQR 10–14), respectively. Of the 150 patients who failed prior thiopurine therapy due to intolerance, 112 patients (75%) did not experience any intolerances while on TG. Of these 150 patients, 92 patients (61%) were still using TG at final follow-up. Furthermore, of the 18 patients (9%) who were primary (*n* = 12) or secondary (*n* = 6) resistant to conventional thiopurines, 14 patients (78%) had a positive response to TG at 12 months. Of the primary resistant patients, nine out of 12 (75%) were responding to TG at 12 months.

### Safety

Seventy-one adverse events occurred in a total of 28% (*n* = 54) of patients. Overall, 22% of patients had to stop TG therapy due to intolerance. No deaths occurred in our cohort study related to TG therapy. Adverse events to TG are shown in Table 2. Laboratory abnormalities occurred in 13% (elevated liver function tests or myelotoxicity) and signs/symptoms in 23% (mainly rash). Pancreatitis occurred in 1 of 30 patients with a history of conventional thiopurine-induced pancreatitis. Malignancies occurred in three patients (2%): two patients developed moderate graded (i.e. requiring minimal, local or non-invasive intervention; melanoma and recurrent breast cancer) and one patient severe graded malignancy (diffuse large B-cell lymphoma). The latter was diagnosed on surgical specimen following excision of enterocutaneous fistula and redo of ileocolic anastomosis after 4 years of TG treatment. This patient was previously treated with both anti-TNF therapy and methotrexate.

**Table 2:** Adverse events (*n* = 71) during a median follow up of 36 months of TG therapy

	Grade 1 (mild) (***n*** = 59)		Grade 2 (moderate) (***n*** = 9)		Grade 3 (severe) (***n*** = 3)	
**General, neurological and psychological**	Flu-like symptoms	7	–		–	
**Blood, lymphatic and vascular disorders**	Bone marrow suppression	12	Bone-marrow suppression	2	–	
**Musculoskeletal and skin disorders**	Alopecia	5	–		–	
	Rash	9				
	Arthralgia/myalgia	8				
**Hepatobiliary disorders**	DILI	9	DILI	3	Portal hypertension	2
**Gastrointestinal disorders**	Nausea	5	Diarrhea	1	Pancreatitis	1
	Abdominal pain	4				
**Infections and infestations**	–		EBV infection	1	Herpetic eye infection	1
**Neoplasm benign and malign**	–		Melanoma	1	Diffuse large B-cell lymphoma	1
			Recurrent breast cancer	1		

*DILI* Drug-induced liver injury, DILI grade 1 is defined as alanine aminotransferase (*ALT*) and aspartate aminotransferase (*AST*) > 1.25 and ≤ 3 upper limit of normal (*ULN*) and total bilirubin > 1.25 and ≤ 2 ULN, grade 2 is defined as ALT and AST > 3 and ≤ 5 ULN and total bilirubin > 2 and ≤ 3 ULN. Bone marrow suppression: grade 1 < =25% reduction from normal cellularity for age, grade 2: > 25 - < 50% reduction from normal cellularity for age, grade 3: > 50 - < =75% reduction cellularity from normal for age

Non-cirrhotic portal hypertension (NCPHT) occurred in two patients (1%). The first patient was between 50 and 60 years old and diagnosed with Crohn’s disease who started TG therapy in 2011 after poor response to both AZA and low dose AZA with allopurinol afterwards. TG was initiated at 20 mg twice daily (40 mg/day) and gradually reduced to 20 mg on alternate days with occasional complete breaks in treatment. In January 2016, the TG was stopped after the patient had been established on anti-TNF therapy for 12 months combined with recurrent episodes of pancytopenia. Anti-TNF therapy was ceased in July 2016 due to loss of response and a severe psoriatic reaction. In December 2017, the patient was admitted with ascites and features of portal hypertension. Investigations (within 8 weeks) determined normal portal pressures and a liver biopsy was reported as normal. The further work up for chronic liver disease and coagulopathy was negative. A clinical diagnosis on NCPHT was established and therapy was discontinued. Symptoms improved with conservative measures such as spironolactone treatment.

The second case of NCPHT was between 60 and 70 years old, diagnosed with Crohn’s disease and had three ileocecal resections prior to using TG. TG was started after not tolerating AZA and failure of both infliximab and adalimumab. Azathioprine was discontinued because of mild fibrosis and steatosis observed in the liver biopsy performed due to abnormal liver enzymes. During TG therapy, no follow-up liver biopsy was performed. After using TG 20 mg twice daily (40 mg/day) for 5 years, the patient presented with abdominal pain. Gastroscopy revealed gastric fundal varices and a CT-scan showed widespread intra-abdominal non-haemorrhagic varices and small volume ascites. TG was discontinued and the patient was given atenolol for primary prevention of variceal bleeding. No varices bleeding occurred over time.

### Biochemistry

Laboratory parameters at initiation of therapy and 6 months of treatment are depicted in Table 3. Among the entire cohort, alanine transaminase (ALAT) and alkaline phosphatase (AP) levels prior to TG initiation did not differ from levels at 6 months. However, a statistically significant increase in bilirubin concentration (8 to 9 μmol/L) and a decrease in platelets (313 to 275 × 10^9^/L) was observed (*p* < 0.05). The platelet level was lower in the response group compared to the non-response group but still within the normal range (259 versus 305 × 10^9^/L, *p* < 0.05). Regarding efficacy, a biochemical response at 6 months was observed in the patients experiencing a positive clinical response as their CRP level was statistically significantly lower compared to the non-responders (9 versus 26 mg/L, *p* = 0.02). There was no statistical difference in erythrocyte sedimentation rate (ESR) level between responders and non-responders (*P* > 0.05).

**Table 3:** Laboratory parameters at initiation of TG (T_0_) and 6 months of treatment (T_6_), shown as mean (SD). Levels of response and non-response group at T_6_ are compared, as are levels at T_0_ and T_6_ of the entire cohort

Parameter	T_**0**_, Response	T_**6**_, Response	T_**0**_, Non-Response	T_**6**_, Non-Response	T_**0**_, Total	T_**6**_, Total
**Haemoglobin**	131 (14)	136 (17)*	128 (21)	132 (16)*	131 (16)	135 (18)*
**Platelets**	305 (87)	259 (95)*	332 (116)	309 (90)	313 (95)	275 (97)*
**Bilirubin**	8 (6)	9 (9)	7 (4)	8 (4)	8 (6)	9 (8)*
**ALAT**	24 (19)	25 (14)	20 (12)	20 (14)	23 (17)	24 (14)
**AP**	74 (30)	81 (38)	90 (31)	94 (58)	79 (32)	84 (41)
**Albumin**	44 (5)	44 (5)	44 (4)	44 (3)	44 (4)	44 (5)
**CRP**	12 (32)	9 (20)	9 (11)	26 (65)*	11 (29)	13 (36)
**ESR**	17 (20)	13 (12)	18 (15)	17 (14)	17 (16)	15 (12)

*ALAT* Alanine aminotransferase, *AP* Alkaline phosphatase, *CRP* C-reactive protein, *ESR* Erythrocyte sedimentation rate

*: statistical significantly different (*P* < 0.05)

TPMT measurements were performed in 87 patients (45%), the median TPMT level was 47 mu/L (IQR 34.5–96). In total, three patients (2%) had low TPMT activity (< 25 mu/L) and 37 patients (19%) had high TPMT activity (> 65 mu/L). All patients in the low activity group suffered from mild side-effects (e.g. myalgias, nausea and rash) on thiopurines. In the high TPMT activity group, 49% of patients discontinued TG, while in the normal TPMT activity group 32% of patients discontinued TG during follow-up (*P* = 0.13). However, if a cut-off level of 44 mu/L (based on the rate of TG discontinuation) was used, 54% of patients above this cut-off level discontinued TG while 23% of patients below this level discontinued TG (*P* = 0.01, Chi-Square test). Eleven patients above this cut-off value stopped TG due to non-response, while no patients stopped due to non-response under this cut-off level (*P* = 0.001, Chi Square test). In these 11 patients, the mean TPMT level was 91.6 ± 26.9 mu/L.

**Fig. 3:**
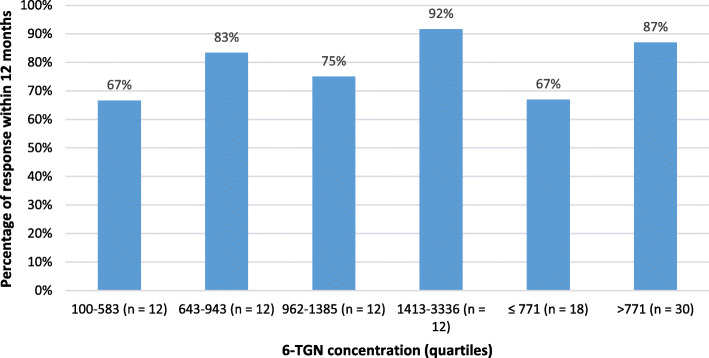
Correlation between 6-TGN concentration and clinical response rate within 12 months. No significant difference was found between the four quartiles (*p* = 0.47, Pearson Chi Square). At a cut-off level of 771 pmol/8 × 105 RBC no significant difference was found below and above the cut-off level (*P* = 0.09, T-test)

Median steady-state 6-TGN concentrations during the first 12 months of TG therapy were available in 48 patients (25%). The median 6-TGN concentration was 953 pmol/8 × 10^5^ RBC (IQR 145–1761). Median 6-TGN metabolite levels were not significantly higher in clinical responders compared to non-responders (966 [IQR 705–1489] versus 764 [IQR 418–1265], *p* = 0.32). No significant differences were found between quartile 6-TGN levels (Fig. 3, *p* = 0.47). The response rate at 12 months was 67% in patients with a 6-TGN cut-off level below 771 pmol/8 × 10^5^ RBC, whereas the response rate was 87% in patients with a cut-off above 771 pmol/8 × 10^5^ RBC. This difference was not statistically significant.

### Radiology and liver biopsy

Imaging and/or biopsies of the liver were performed in case of suspicion of NCPHT or as part of routine screening. An ultrasound was performed in 58 patients (30%) at a median of 28 months (IQR 14–41). Thirty-six patients (62%) had no abnormalities, 9 (16%) borderline splenomegaly, 16 (28%) signs of steatosis and one (2%) other unrelated abnormalities. Four patients demonstrated both steatosis and splenomegaly. Eighty-three patients (43%) underwent an magnetic resonance imaging (MRI) at a median follow-up of 37 months (IQR 21–60), showing no abnormalities in 58 patients (70%), borderline splenomegaly in seven (8%), steatosis in nine (11%), focal nodular hyperplasia in one (1%), hepatomegaly in 2 (2%) and other abnormalities (all liver cysts) in seven (8%).

Liver biopsies were performed in 33 patients (17%) during TG treatment, at a median follow-up of 18 months (IQR 11–31). NRH was histologically diagnosed in two patients, signs of possible NRH were found in five patients and normal histology was found in the remaining 26 patients. One NRH patient used 20 mg once daily, the other NRH patient 20 mg twice daily. All five patients with signs of NRH were started on 40 mg/day, which was reduced to 20 mg on alternate days in one patient. The first NRH patient, also diagnosed with primary sclerosing cholangitis, suffered from varices grade I on gastroscopy for which this patient underwent band ligation. NRH was detected 5 months after cessation of TG, which had been used for 10 months. The second NRH patient was asymptomatic and developed NRH after 17 months of TG therapy. Both patients with NRH had a history of methotrexate therapy and side-effects on thiopurines.

All five patients with signs of NRH on liver biopsy were asymptomatic and did not develop clinical features of NCPHT during follow-up. Four of those five (80%) were able to continue TG until final follow-up. Nine of the 14 patients (64%) demonstrating abnormalities (spleno- or hepatomegaly) on ultrasound and/or MRI underwent a liver biopsy. Three patients of the remaining five patients who did not receive a liver biopsy were still using TG at final follow-up without signs of NCPHT. Two remaining patients who did not receive liver biopsy discontinued TG. One patient discontinued due to persistent thrombocytopenia and elevated liver function enzymes, and the other patient (known with cirrhotic liver disease) discontinued because of elevated liver enzymes and perisplenic and gastro-oesophageal varices. The latter had portal hypertension which developed due to postoperative portal vein thrombosis after a right hemicolectomy, which was performed 2 years before TG initiation.

## Discussion

This retrospective, multicentre study sought to look at the real-world outcomes of TG from three large UK IBD units (over 4000 IBD patients per unit). A complete data capture was achieved through a search of local TG databases (prescriptions and IBD databases). This yielded 193 patients with a median follow up of 3 years and a median dose of 20 mg/day. Thioguanine can be started at its therapeutic dose as it is well tolerated, resulting in a positive clinical response of 71% at 6 months and 65% at 12 months as demonstrated in our study. Following this initial positive response, most patients continued to tolerate the treatment long-term with an overall efficacy at final follow-up of 54%. In our study, no correlation was found between 6-TGN levels and clinical response.

Meijer et al. published a systematic review on the efficacy of TG in IBD. They reported results of 11 studies, comprising of 353 patients who were treated with a median TG starting dose of 20 mg/day after failing prior conventional thiopurine therapy. Clinical improvement in terms of decreased disease activity score or the ability to cease/decrease corticosteroid use was observed in 65% of patients (*n* = 228) [34]. Recently, Simsek et al. published the largest TG cohort consisting of 274 IBD patients, with a long-term follow-up of 4 years. They found that TG was tolerated in 79% of patients, 66% was responding at 6 months and 51% showed sustained clinical effectiveness. Although 40% of patients developed adverse events (65% mild, 30% moderate and 5% severe), only 11% overall discontinued due to intolerance. These authors also provided a meta-analysis of 483 IBD patients on at least 12 months of TG treatment. They reported that 231 patients achieved a positive clinical effect (pooled proportion: 44, 95% CI: 34–55%) [14]. The results described above are broadly in line with the outcomes of our cohort.

In our study there were two patients with histological features of NRH in their liver biopsies. Five patients had histological characteristics suspicious of NRH during liver biopsy, but none of them developed clinical signs of NCPHT. Only one patient, who did not respond to AZA and AZA-allo, discontinued TG during follow-up also due to poor-response. Additionally, two other patients (1%) were diagnosed with NCPHT in this cohort, without NRH-related characteristics in their liver biopsies. As compared to historical data from Dubinsky et al. (2003) in which high rates of NRH were described: 16 of 26 liver biopsies demonstrated features of NRH (62%), our numbers are comparable to the background incidence of NRH (6%). The worrying high NRH rate in Dubinsky’s study is not completely understood, but may be associated with the used higher doses of TG (median 45 mg/day, with an undocumented doses in the subgroup of patients who developed liver toxicity) and/or a selection bias as TG was used in patients with hepatotoxicity during AZA/MP therapy [35].

More recently, van Asseldonk et al. assessed the occurrence rate of NRH during TG therapy in 111 IBD patients. These patients had at least one liver biopsy, a mean daily TG dose of 21 mg (SD 5 mg) and a median treatment duration of 20 months. In this group only seven patients (6%) were histologically diagnosed with NRH and none had complications [36]. Ward et al. evaluated 54 patients treated with a mean TG dose of 27 mg/day in a group of patients intolerant or resistant to conventional thiopurines or methotrexate. No NRH was observed in their study [16]. Pavlidis et al. investigated a similar sized cohort of 62 IBD patients who received a median TG dose of 20 mg twice daily. The findings included mild NRH in one patient who was procoagulant due to antiphospholipid syndrome (1.6%), resulting in a portal vein thrombosis with complications. This group also had a patient with signs of NRH that resolved after dose reduction of TG to 20 mg once daily [15]. To determine the background incidence of NRH, de Boer et al. did 83 liver biopsies (during gastrointestinal surgery) in a thiopurine-naive IBD cohort and detected a NHR prevalence of 6% [37]. Furthermore, Wanless et al*,* reported 64 cases (2.6%) of NRH among 2500 consecutive autopsies in the general population [38]. These observations suggest that patients treated with low dose TG (not exceeding 25 mg/day) do not have a considerable higher risk of developing NRH when compared to non-TG receiving patients. Furthermore, it is important to note that the conventional thiopurines (AZA/MP) can cause NRH as well and were used in all our patients [39, 40]. Moreover, both of our NRH cases in our study were previously treated with methotrexate and one was also diagnosed with primary sclerosing cholangitis. Regarding the two NCPHT patients, one of them presented with signs of NCPHT (with a normal liver biopsy) almost 2 years after TG cessation. The other patient had already steatosis and fibrosis on liver biopsy while still on AZA. It is therefore important to state that we cannot determine which of the NRH and NCPHT patients of our study may have actually developed NRH and NCPHT due to other causes.

Furthermore, van Asseldonk et al. demonstrated that although vascular liver abnormalities of any degree are commonly observed (27%) in TG treated IBD patients, they rarely become progressive or are of any clinical relevance [41, 42]. It was found by Morris et al. that NRH survival is highly variable and mostly related to age and underlying disease [43]. Seven patients in this study withdrew TG due to pregnancy, as the current UK guidelines do not allow this treatment in pregnancy, as compared to AZA/MP. Theoretically, there should be no difference between TG and AZA/MP. Thiopurines are not associated with any congenital abnormalities in men or women [44, 45]. Although data about safety of TG during pregnancy is scare, there is no evidence demonstrating negative effects. There is one case series published on 19 pregnancies in 13 IBD patients who were treated with low dose TG (20 mg/day). All patients conceived healthy new-borns, supporting the safe use of TG during pregnancy [46].

To our knowledge, the present study is the second largest study evaluating the safety and efficacy of TG in IBD patients. Patients from three different hospitals were included, which created the possibility of assessing slightly different low-dose TG treatment regimens. Secondly, the patients of our study have a long follow-up period, allowing us to assess the long-term effects of TG treatment. As a result of the retrospective design of the study, we acknowledge a few limitations. Firstly, it was difficult to compare the laboratory and imaging results between patients, as these were not regularly performed at fixed time intervals. Secondly, splenomegaly, hepatomegaly and signs of NRH were not defined using a standardised definition, therefore it was complicated to objectify and compare these parameters. Thirdly, it was not possible to assess mucosal response and clinical symptoms, as too limited endoscopic reports and disease activity indices (Simple Clinical Colitis Activity Index or Harvey-Bradshaw Index) were available. Furthermore, faecal calprotectin was not routinely measured and therefore not evaluated by us. Lastly, 6- TGN and TPMT levels were not available in the majority of our patients what led to calculating the correlation of 6-TGN level and clinical response using a relatively small number of patients. Also, the 6-TGN data is likely to be affected by selection bias as metabolite levels are mostly performed in patients having an insufficient response to TG. A 6-TGN cut-off level above 700 pmol/8 × 108 RBC seems to be associated with better clinical effectiveness [14]. In our study, patients having 6-TGN levels above 771 pmol/8 × 10^5^ RBC had a response rate of 87%, while patients who had 6-TGN levels under this cut-off level had a response rate of 67%. This difference in response, although not statistically significant, might suggest that 6-TGN levels above 700 pmol/8 × 108 RBC can be associated with better clinical effectiveness as demonstrated by Simsek et al. [14]. One of the causes of low 6-TGN levels might be the result of drug incompliance. So, 6-TGN levels can be used to assess drug compliance in IBD patients treated with TG. Furthermore, 11 out of 15 patients who stopped TG due to non-response had TPMT levels above the cut-off level of 44 mu/L. This is in line with results that were found in literature for AZA treated IBD patients.

Ansari et al. [47] found that high TPMT activity (> 14 u/mL) was significantly associated with non-response in AZA-treated IBD patients (OR 0.21, 95% CI 0.07–0.68). It seems that high TPMT activity might also be associated with non-response in TG-treated IBD patients. Although, the numbers in this study are small, it might indicate that further optimization of TG dosing might be useful by measuring the TPMT level.

The outcomes of this study are in a subset of difficult-to-treat patients who all have failed prior thiopurine therapy. Moreover, a substantial number of patients in our study were already unsuccessfully treated with biologicals and/or allopurinol co-therapy (40 and 39% respectively). Still, in this heavily treated group of patients, TG has shown to be an effective and well-tolerated therapy. A prospective study is needed to establish the role of TG as a rescue therapy for IBD patients who fail conventional thiopurine or other immunomodulation therapy (biological therapy, AZA-allo, methotrexate, tacrolimus, tofacitinib), and as first-line maintenance therapy for IBD.

## Conclusions

Long-term follow-up in this English cohort of 193 difficult-to-treat IBD patients suggests that low-dose TG (≤ 40 mg/day) can be effective and well-tolerated in more than half of patients who had failed prior thiopurine therapy and other immunomodulation or biological therapy. Hepatotoxicity was not common and the incidence rate of NRH was similar to the background incidence of NRH in IBD. There were no differences between 20 and 40 mg/day in terms of efficacy and safety, but we recommend to use the lowest possible effective dose of 20 mg/day or lower. A prospective trial is recommended to further establish the role of TG in IBD patients as first-line or rescue treatment.

## Data Availability

The datasets generated and/or analysed during the current study are not publicly available but are available from the corresponding author on reasonable request.

## Abbreviations

NRH: Nodular regenerative hyperplasia
TG: Thioguanine
IBD: Inflammatory bowel disease
AZA: Azathioprine
MP: Mercaptopurine
CD: Crohn’s disease
UC: Ulcerative colitis
6-TGN: 6-thioguanine nucleotides
