# Correction to: Efficacy, safety and drug survival of thioguanine as maintenance treatment for inflammatory bowel disease: a retrospective multi-centre study in the United Kingdom

**DOI:** 10.1186/s12876-021-01992-2

**Published:** 2022-04-26

**Authors:** Ahmed B. Bayoumy, Elsa L. S. A. van Liere, Melek Simsek, Ben Warner, Aathavan Loganayagam, Jeremy D. Sanderson, Simon Anderson, Jonathan Nolan, Nanne K. de Boer, Chris J. J. Mulder, Azhar Ansari

**Affiliations:** 1 Department of Gastroenterology and Hepatology, Amsterdam UMC, VU University Medical Centre, Amsterdam, The Netherlands; 2 Department of Gastroenterology, Guy’s and St Thomas’ NHS Foundation Trust, London, UK; 3 Department of Gastroenterology, Queen Elizabeth Hospital, Woolwich, UK; 4 Department of Gastroenterology, Surrey and Sussex NHS, Easy Surrey Hospital, Surrey, UK; 5 Department of Gastroenterology and Hepatology, Amsterdam UMC, VU University Medical Centre, AG&M Research Institute, Amsterdam, The Netherlands

## Correction to: BMC Gastroenterology (2020) 20:296 10.1186/s12876-020-01441-6

After publication of this article [1], the authors reported three adjustments:

1. Competing interests: the travel grants were paid by HLW Pharma instead of TEVA Pharma.
2. TGN-units all over the manuscript have to be adjusted from pmol/8 × 10^5^ RBC to pmol/8 × 10^8^ RBC.
3. Some minor textual adjustments.

The original article [1] has been updated.
